# The relationship between deltamethrin-induced behavioral changes and acetylcholinesterase activity in zebrafish embryos or larvae based on transcriptome

**DOI:** 10.3389/fvets.2024.1526705

**Published:** 2025-01-23

**Authors:** Chunyu Liu, Yantong Guo, Xue Zhang, Hongsong Chen, Maomao Han, Han Wang, Jieyao Niu, Jinfei He, Jingfeng Yang, Wu Dong, Jiangdong Xue

**Affiliations:** College of Animal Science and Technology, Inner Mongolia Minzu University, Tongliao, China

**Keywords:** deltamethrin, zebrafish embryos, neurotoxicity, behavioral changes, acetylcholinesterase

## Abstract

**Background:**

Deltamethrin (DM) is a broad-spectrum insecticide that is widely used to control agricultural pests. Recently, DM has posed a potential threat to the health of infants and young children, and this is because of the environmental and food pollution that is caused by the extensive use of DM.

**Methods:**

In this study, zebrafish (*Danio rerio*) embryos were used as experimental animals to quantify the behavioral changes of larvae induced by DM and explore the relationship between DM and acetylcholinesterase activity.

**Results:**

The results showed that DM caused an increase in coiling movement, heart rate, and apoptosis in the brain in early zebrafish embryos or larvae. It also caused a decrease in the expression of acetylcholinesterase-associated genes and the activity of acetylcholinesterase, which also led to an increase in the acetylcholinesterase content. The transcriptome data also showed that low concentration DM induced acetylcholine-related gene signaling pathways. The above results suggest that low doses of DM may induce neurodegeneration because DM exposure inhibits acetylcholinesterase, leading to brain cell apoptosis and behavioral changes in the zebrafish embryos or larvae. Micro-injection of zebrafish embryos at the 2–4 cell stage knocked down or overexpressed the acetylcholinesterase gene showed that the behavior and enzyme activity of zebrafish had some effects.

**Conclusion:**

This study explored the relationship between acetylcholinesterase and the change in zebrafish behavior caused by DM exposure to provide a basis for the treatment of DM poisoning in the aquaculture.

## 1 Introduction

Pyrethroids were produced in the 18th century and were initially in the form of a crude extract of chrysanthemum (1, 40). Currently, pyrethroid insecticides have become one of the most widely used insecticides worldwide, making up about a quarter of global insecticide usage (2). Additionally, they also cause extensive residues of pyrethroids, which seriously threaten the health of animals and humans. Among them, deltamethrin (DM) is one of the most widely used pyrethroid insecticides (3, 4). Recently, due to restrictions on organophosphorus pesticides, DM has been more widely used as a substitute. The spraying of pesticides pollutes the aquatic environment through runoff from farmland, and DM has also become a common source of pollution in aquatic systems (5, 39).

Animals that are exposed to DM exhibit a pronounced central nervous system syndrome. When rats were exposed to DM, they first experienced restlessness, respiratory distress, and severe convulsions. After 3–5 h, the rats showed symptoms, such as choreoathetosis, loss of the righting reflex, and tonic-clonic convulsions (6, 7, 41). Though DM has been reported to be less toxic to mammals and birds, it has also been shown to be highly toxic to aquatic organisms, especially fish, DM exposure caused edema and neurotoxicity in fish, delayed egg formation, delayed embryonic development, larval deformities, and reduced villi surface tension (8–10). Thus, the use and residue of DM will eventually pose a threat to human health via the food chain.

Recent findings suggest that acetylcholinesterase (AChE) is the main target enzymes for the toxic effects of pyrethroids (11–14, 42). Specifically, AChE plays a key role in the biological neural signal transduction process. Its core physiological function is to hydrolyze acetylcholine, end the stimulating effect of this neurotransmitter on the postsynaptic membrane, so as to ensure the normal conduction of nerves and maintain the stable state of the nervous system. And they found that AChE activity in different tissues of zebrafish (*Danio rerio*) decreased with the increase of DM exposure concentration. The activity of acetylcholinesterase decreased with the extension of exposure time. In different tissues, the inhibition of AChE at a sublethal dose shows a lag, and the chemical first inhibits AChE in the brain (15). Fenvalerate exposure was found to have a strong inhibitory effect on AChE activity in *Cirrhinus mrigala*, leading to a large accumulation of ACh in the fish and causing abnormal behavior and physiological functions and eventually leading to death (16). Acetylcholine esterase activity and the ACh content have been used as specific biomarkers for pyrethroid pesticides (17–19).

Zebrafish have strong adaptability, a low feeding cost, a short reproductive cycle, and abundant biological information available (20). Currently, zebrafish have become one of the most important vertebrate models for studying embryonic development and the neural circuit (21). In this study, zebrafish embryos were used as a model to study the neurotoxicity and mechanism of DM in zebrafish by detecting changes in morphology, behavior, histology, and gene and protein levels.

## 2 Materials and methods

### 2.1 Experimental animals

Zebrafish (AB line) were provided by the Inner Mongolia Key Laboratory of Toxicant Monitoring and Toxicology and raised in a circulating aquaculture system (Beijing Aisheng Science and Technology Development Co., Ltd.). The rearing conditions were as follows: the male and female fish were housed in separate tanks and fed worms twice a day in the morning and evening; the temperature was maintained at 28.0–28.6°C under a light-dark cycle of 14:10 h; the pH remained between 7.0 and 7.6 and the conductivity remained between 440 and 640 μs. When breeding, the male and female adults were combined in a 2:1 ratio. The embryos were collected and washed the next morning. Then, embryo selection was performed under a Leica stereomicroscope (Leica, M205, Singapore).

### 2.2 Experimental reagents

Deltamethrin, (C_22_H_19_BrNO_3_, purity >99.2%) was purchased from J&K Scientific (Beijing, China). Acetylcholinesterase activity assays were purchased from Solaibo Technology Co., Ltd. (Beijing, China). An ACh Content Test Kit, and BCA Protein Quantitation Kit were purchased from Nanjing Jiancheng Bioengineering Institute (Nanjing, China). Then, dimethyl sulfoxide (DMSO), total RNA extraction reagents, and other reagents were purchased from Sigma (St. Louis, MO, United States). The reverse transcription reagents were purchased from Takara (Kusatsu, Japan). A terminal deoxynucleotidyl transferase dUTP nick end labeling (TUNEL) staining kit was purchased from Beyotime Reagent Co., Ltd. (Beijing, China).

### 2.3 Zebrafish embryos exposure to deltamethrin

Zebrafish embryos (2–4 hpf, hours post fertilization) were selected and exposed with/without DM (0.1% DMSO and 0.05 μg/L, 0.5 μg/L or 5 μg/L) up to 120 hpf, and there were 10 embryos per group and three parallel replicate groups. Every 24 h, the morphological changes were detected, such as the mortality, hatching rate, and deformity rate, and the heart rate was detected at 72, 96, and 120 hpf. Moreover, behavioral tests were conducted at 120 hpf and the embryos were collected for tissue sections and the detection of gene and protein levels.

### 2.4 Behavioral testing

For the coiling frequency detection, the zebrafish embryos were exposed with/without DM (0.1% DMSO and 0.05 μg/L, 0.5 μg/L, or 5 μg/L) from 2–4 until 32 hpf following the method of Chen et al. (22). The coiling frequency of the zebrafish embryos was recorded for 1 min every 2 h from 18 to 32 hpf under a Leica stereomicroscope. There were 10 zebrafish larvae in each group and three parallel experimental groups.

For the 120 hpf zebrafish behavioral assays, the zebrafish embryos were exposed with/without DM (0.1% DMSO and 0.05 μg/L, 0.5 μg/L, or 5 μg/L) from 2–4 until 120 hpf with the Viewpoint Zebra Lab Behavior System (Viewpoint, Civrieux, France) according to the method of Zhou et al. (23). For each experimental group, 24 well-developed zebrafish larvae without deformity were selected. Subsequently, the zebrafish larvae were placed in a 24-well plate that was filled with 2 mL of fish solution per well. Then, we waited 10 min for zebrafish acclimatization, and the fish were subjected to 10 min of alternating light and dark conditions to detect any zebrafish behavioral changes for 60 min in total. The output consisted of one data point per minute for the statistical analysis.

### 2.5 Terminal deoxynucleotidyl transferase dUTP nick end labeling staining

When the zebrafish embryos developed to 120 hpf, the zebrafish larvae were collected, immobilized with 4% PFA, washed with PBS, dehydrated with a series of alcohols, and paraffin-embedded. Then, 3 μm sections were cut, the sections were dewaxed twice in xylene at 10 min/time, and they were dehydrated with a series of alcohols. After washing with water, the sections were digested with protein kinase K at 37°C for 15 min and PBS was washed twice for 5 min each time. Next, according to the instructions, TUNEL fluorescent dye (Beyotime Reagent Co., Ltd.) was dropped onto the sections, and they were placed in a thermostatic oven at 37°C in the dark. Subsequently, the sections were washed with PBS, dried, and sealed.

### 2.6 Extraction of the total RNA and relative quantification of the gene expression

The zebrafish embryos (2–4 hpf) were selected and exposed with/without DM (0.1% DMSO and 0.05 μg/L, 0.5 μg/L or 5 μg/L) up to 120 hpf with 10 embryos per group, and three parallel replicate groups. The zebrafish larvae were collected at 120 hpf, and the expressions of *myh6*, *myh7*, *chrnb3b*, and *ache* messenger RNA (mRNA) were quantified. According to the manufacturer’s instructions, the total RNA was extracted with Trizol reagent. Then, the RNA concentrations were measured using the NanoDrop ND-100 spectrophotometer (Thermo Fischer Scientific, Waltham, MA, United States), which was used to ensure that the 260/280 ratios were greater than 1.8. Next, high-capacity complementary DNA (cDNA) reverse transcription kits (Takara) were used for cDNA generation. Subsequently, 0.25 ng of cDNA was used for a one-step reverse transcription polymerase chain reaction (PCR) reaction (Takara). The samples were analyzed using the Plus One Real-time PCR system (AB Biosynthesis). Then, *myh6*, *myh7*, *chrnb3b*, and *ache* were studied, and *gapdh* was used as an internal reference. The primer sequences are presented in Table 1, and the relative gene expression was calculated using the $2^{-\Delta \Delta C_{T}}$ method.

**Table 1:** Primer sequences for reverse transcription polymerase chain reaction.

Primer	Sequence (5′–3′)	Length/bp
*Myh6*	F: GCTGATAGCGACCGATAAGG	176
	R: TATCTGCGTCTTCAGTGCCG	
*Myh7*	F: CTGGAGGACGAATGCTCTGA	205
	R: CCAGTGTCTGCTGATGAGCT	
*Chrnb3b*	F: TGGAGCAGGCCACTAACTCT	203
	R: AGGTGGAGGAGTGCTCAGAA	
*AChE*	F: CTACGCCCAGACCACAGAAC	103
	R: GCCAGGTAACGGCCATCATA	
*Gapdh*	F: GCTCACATTAAGGGTGGTGC	137
	R: TTGGTGGTGCAGGAGGCATT	

### 2.7 Transcriptome analysis

Zebrafish embryos were treated with 0.1% DMSO and 0.5 μg/L or 5 μg/L DM, and transcriptomic analysis was performed in the range of 2–3 to 120 hpf, with 15 zebrafish in each treatment group. There were 3 replicates in each group, and total RNA was extracted with Trizol reagent (*n* = 3). RNA sequencing (RNA-SEQ) was then performed by Novo Ho (Tianjin, China). Genes with adjusted *p*-value (*p*adj) <0.05 and fold change >1.5 were defined as differentially expressed genes (DEGs). Next, the Kyoto Encyclopedia of Genes and Genomes (KEGG) was used to analyze the major enrichment pathways of differential genes using R 3.2.2. And the Novo Ho digital analysis tool was used for Gene Ontology (GO) enrichment analysis. Significantly enriched GO terms These GO terms can analyze gene changes in biological processes (BP), cell components (CC) and molecular functions (MF). The corrected *p*-value (*p*adj) <0.05 was significant.

### 2.8 Acetylcholinesterase activity and acetylcholine content assays

The zebrafish larvae were treated with/without DM from 2–4 until 120 hpf, there were 120 larvae per group with three parallel replicates for each concentration group. The 120 hpf zebrafish larvae were homogenized to extract the proteins.

According to the method of Chen et al. (24), the detection of the AChE activity and ACh content were carried out. The 120 hpf zebrafish larvae were homogenized to extract the proteins, and detection of the AChE activity and ACh content was performed (Solelbro Reagent). The determination of the AChE activity was based on a chromogenic reaction that converts ACh to the blue product trinitrobenzene. Detection of AChE activity was conducted under a 412 nm light wavelength, and then the AChE activity was calculated. The detection of the ACh content was conducted under 550 nm light wavelength, and then the content of ACh was calculated.

### 2.9 Preparing the acetylcholinesterase messenger RNA and *AChE*-MO

According to the methods of Chen et al. (24), AChE mRNA (BC163898.1) was produced by Bgi Tech Solutions Co., Ltd. (Beijing, China), amplified, and synthesized for gene overexpression. The AChE-MO was synthesized by Gene Tools, LLC (United States) (5′-GGTCTTCATGGCTTCTTTTCACTTG-3′). The best 1–2 cell stage zebrafish embryos were selected for microinjection, and each fish egg was injected with 3 nL. Then, l h after injection, the embryos in good condition were examined and selected for the experiments, and they were placed in an incubator at 28°C for culturing. All the embryos or larvae were observed under the microscope daily, and the dead embryos or larvae were removed.

### 2.10 Statistical analysis

The experimental data were analyzed and displayed using GraphPad Prism 5.0 (GraphPad, California, United States), and the data were expressed as the mean ± standard error. A one-way analysis of variance and Turkey multiplex test were used to analyze the significance of the differences between groups, and *p* < 0.05 was considered a significant difference.

## 3 Results

### 3.1 Effects of deltamethrin exposure on the mortality rate, hatching rate, and deformity rate of zebrafish embryos/larvae

**Figure 1:**
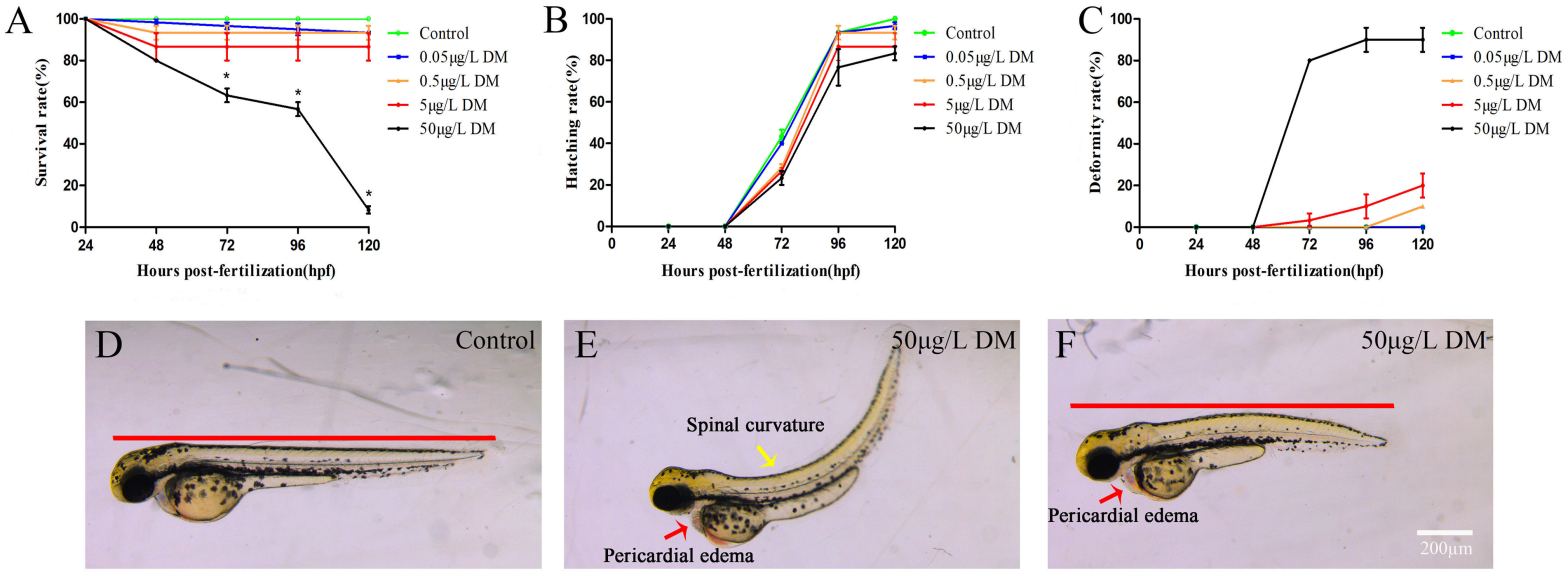
Effects of deltamethrin (DM) exposure on the mortality, hatchability, and malformation rates of zebrafish embryos/larvae. The zebrafish embryos were exposed to DM from 4 to 120 hpf, and the hatching rate, mortality rate, and deformity rate were detected every 24 hpf. **(A)** Mortality rate. **(B)** Hatching rate. **(C)** Deformity rate. **(D)** Morphological photos of zebrafish in 120 hpf control group. **(E,F)** 120 hpf 50 μg/L DM zebrafish morphology photograph (*: *p* < 0.05; **: *p* < 0.01; ***: *p* < 0.001; scale bar = 200 μm).

Zebrafish embryos were exposed to 0.1% DMSO and 2–4 hpf to 120 hpf of 0.05, 0.5, 5, and 50 μg/L DM, and survival, hatching, and malformation of zebrafish were observed and recorded every 24 hpf. The results showed that from 24 hpf to 120 hpf, 0.05 and 0.5 μg/L DM had no significant effects on the mortality and hatchability of zebrafish (*p* > 0.05) (Figures 1A,B). However, 50 μg/L DM caused a significant increase in zebrafish mortality, and at 72 hpf DM with an LC50 of 12.32 μg/L DM also caused significant deformities, such as pericardial edema and spinal curvature. Compared with the control group, the deformity rate was increased by 3.50 times (*p* < 0.05) (Figure 1C). DM also caused significant deformities, mainly manifested as trunk curvature, pericardial edema, and shortened body length, among which pericardial edema was the most significant (Figures 1D–F).

### 3.2 Effects of deltamethrin exposure on the swim bladder and heart sac of the zebrafish

**Figure 2:**
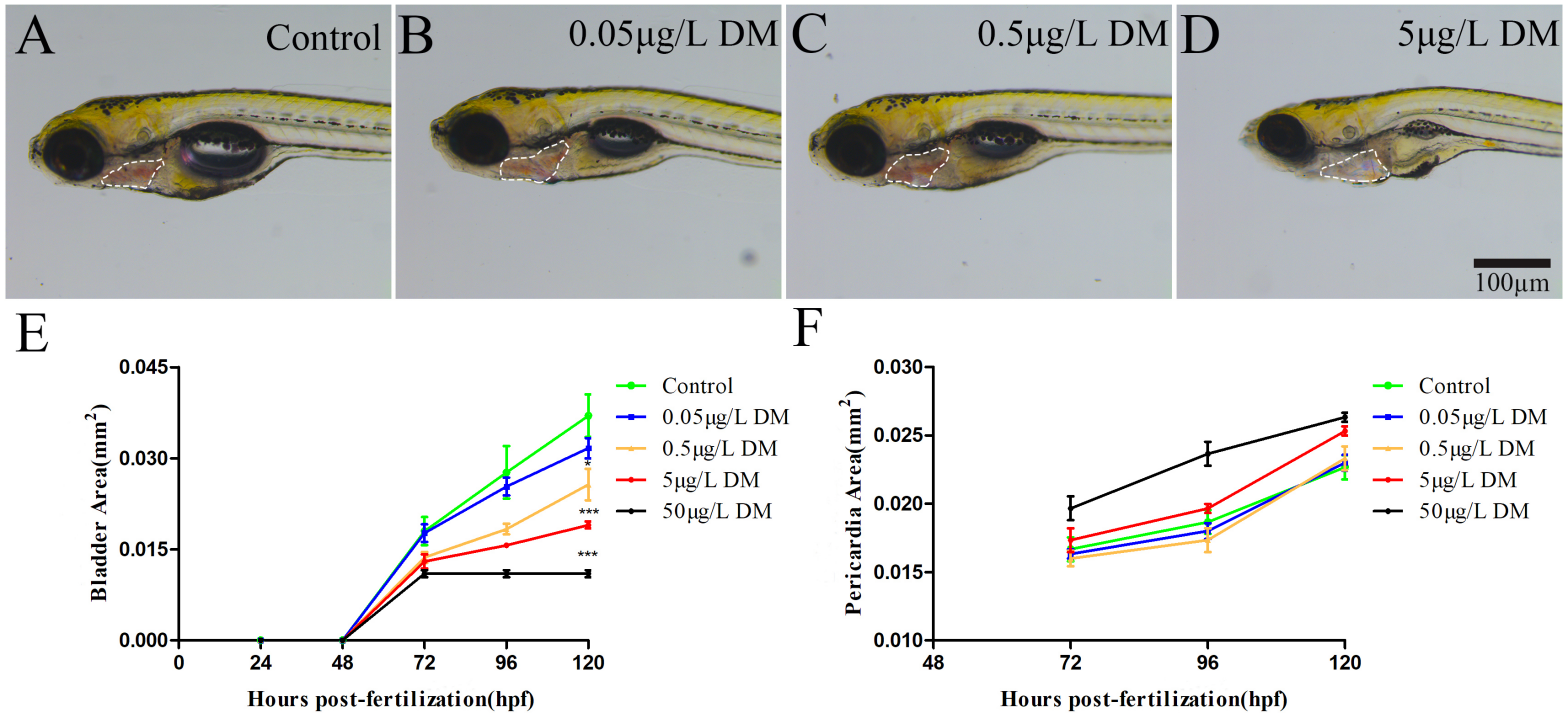
Effects of DM exposure on the development of swim bladder and heart sac in zebrafish. **(A–D)** Photos of young fish. **(E)** Statistics of swim bladder area. **(F)** Cardiac sac area statistics. (*: *p* < 0.05; **: *p* < 0.01; ***: *p* < 0.001; *n* = 10; scale bar = 100 μm).

Zebrafish embryos were exposed to 0.1% DMSO and 2–4 hpf to 120 hpf at 0.05, 0.5, 5, and 50 μg/L DM, at which the development of the zebrafish swim bladder and heart sac was measured and recorded (Figure 2). The results showed that with the increase of exposure concentration, the development of swim bladder of zebrafish in DM exposed group was inhibited. When zebrafish developed to 120 hpf, the swim bladder area of zebrafish in 0.5 and 5 μg/L DM groups was reduced by 30.63 and 48.65%, respectively, compared with the control group (Figure 2E). In contrast, the cardiac sac area increased with the increase of exposure concentration. At 120 hpf, the heart sac area of zebrafish in 0.05, 0.5 and 5 μg/L DM groups increased by 1.12, 1.03 and 1.01 times, respectively, compared with the control group (Figure 2F).

### 3.3 Effects of deltamethrin exposure on behavioral changes in the zebrafish embryos or larvae

**Figure 3:**
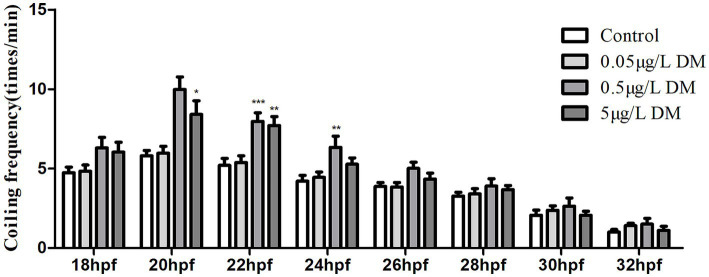
Effect of deltamethrin (DM) exposure on the frequency of the coiling movement in the zebrafish embryos. The zebrafish embryos were exposed with/without DM (at 4 hpf), and the coiling movement frequency of the embryos was detected every 2 h from 18 to 32 hpf (*: *p* < 0.05; **: *p* < 0.01; ***: *p* < 0.001; *n* = 30).

To detect the effect of DM on behavioral changes in early zebrafish embryos, 2–4 hpf zebrafish embryos were exposed to 0.1% DMSO and 0.05, 0.5, 5, and 50 μg/L DM, and the frequency of embryo coiling was detected at 18–32 hpf (Figure 3). When the zebrafish embryo developed to 18 hpf, the zebrafish embryo began to show coiling movement, but DM had no significant effect on the curling movement. However, at 20–22 hpf, 0.05, 0.5, and 5 μg/L DM significantly increased the frequency of embryo coiling by more than 1.45 times compared to the control group (*p* < 0.001). At 24 hpf, the crimp frequency of 0.5 μg/L DM group was significantly increased by 1.51 times compared with the control group (*p* < 0.05). However, 5 μg/L DM did not significantly increase the crimp frequency. At 26–32 hpf, the coiling frequency decreased gradually, but DM exposure had no significant effect on the coiling frequency (*p* > 0.05). 0.05 μg/L DM exposure had no effect significantly on the curled frequency of 18–32 hpf zebrafish embryos (*p* > 0.05).

When detecting the effect of AChE overexpression or AChE knockdown on the behavioral changes in the early zebrafish embryos, the zebrafish embryos (2–4 hpf) were injected with AChE-MO and AChE overexpression and exposed to DM. The frequency of the coiling movement of the embryos was detected at 18–32 hpf (Figure 3). When the zebrafish embryos were at 18 hpf, the coiling movement began to appear, but DM had no significant effect on the coiling movement. However, at 20–22 hpf, 0.5 μg/L DM significantly increased the frequency of the coiling movements, and they reached their highest value relative to that of the control group, with an increase of more than 1.45 times (*p* < 0.05). At 22 hpf, the coiling frequency of the embryos which were injected with AChE overexpression and exposed to 0.5 μg/L DM significantly increased than that in the 0.5 μg/L DM group (*p* < 0.01). At 24–32 hpf, the coiling frequency gradually decreased in each group; when contrasted with the control group, the other four groups did not have significantly affected coiling movements (*p* > 0.05).

**Figure 4:**
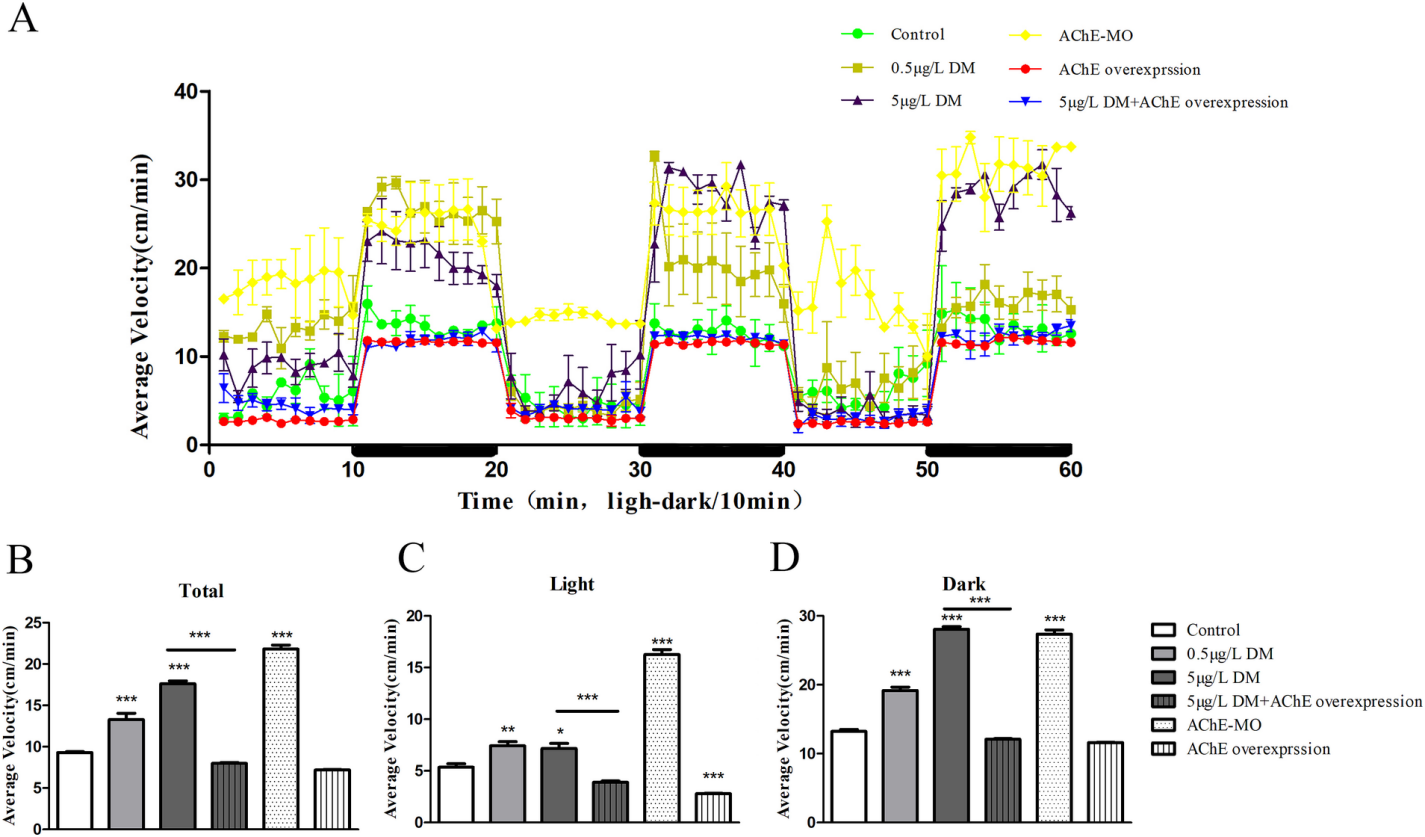
Effect of deltamethrin (DM) exposure on the swimming speed of the zebrafish larvae. The zebrafish embryos were exposed to DM at 120 hpf, behavioral monitoring was performed for 60 min, light and dark conditions were alternated every 10 min, and the average swimming speed per minute for the zebrafish was recorded. **(A)** Linear plot of the swimming distance. **(B)** Total swimming speed. **(C)** Swimming speed in the dark environment. **(D)** Swimming speed in the light environment (*: *p* < 0.05; **: *p* < 0.01; ***: *p* < 0.001; *n* = 12).

When the embryos were at 120 hpf and became larvae, the changes in the swimming speed of the zebrafish larvae were detected under alternating light and dark conditions, and the change index of the one-minute swimming distance was quantitatively analyzed (Figure 4). Specifically, 0.5 μg/L DM and 5 μg/L DM and *AChE MO* increased the swimming speed of the zebrafish larvae under both light and dark conditions in relation to the control group, and the average swimming speed increased by 1.33–2.12 times (*p* < 0.05). In both the light and dark environments, the swimming speed of the embryos that were microinjected with *AChE* and exposed to the 5 μg/L DM group was significantly reduced 56.95 and 45.67% when compared to that of the 5 μg/L DM group (*p* < 0.05; Figures 4A–D).

### 3.4 Deltamethrin exposure increases apoptosis in the brain of zebrafish larvae

**Figure 5:**
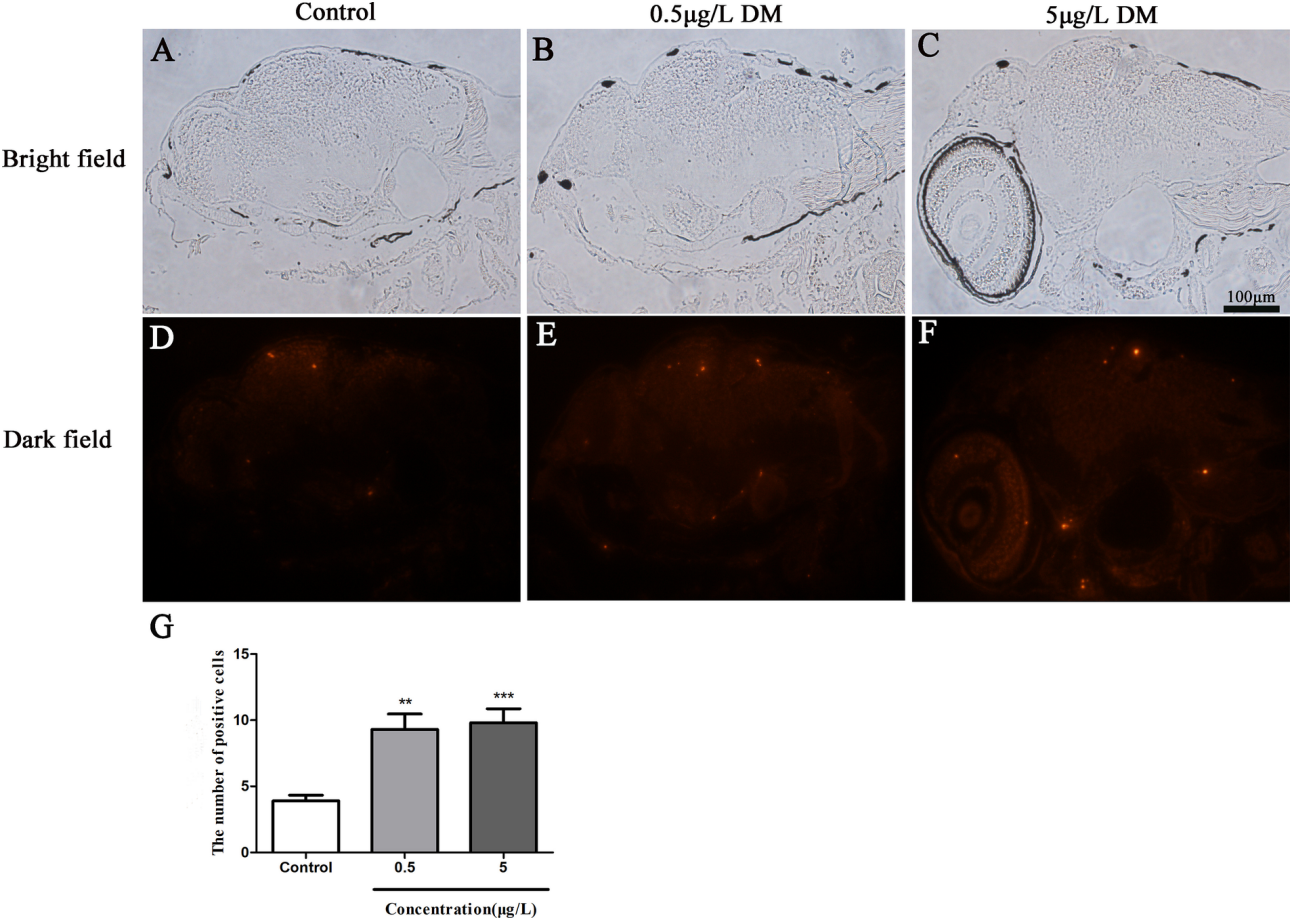
Effects of deltamethrin (DM) exposure on the brain of the zebrafish larvae at 120 hpf. The red indicates the apoptotic fluorescent protein label. **(A–C)** Bright field. **(D–F)** Dark field. **(G)** Results of positive cell number (**: *p* < 0.05; ***: *p* < 0.01; scale bar = 100 μm).

The zebrafish larvae underwent one-step TUNEL staining at 120 hpf (Figures 5A–F). The TUNEL-positive cells were observed in the brain of the zebrafish larvae (Figures 5D–F). The 0.05 and 0.5 μg/L DM treatment significantly increased the number of TUNEL-positive cells. When compared with the control group, the number of TUNEL-positive cells in the 0.05 and 0.5 μg/L DM groups increased by 1.65 and 1.82 times respectively, (*p* < 0.05; *p* < 0.01; Figure 5G).

### 3.5 Transcriptome sequencing analysis

#### 3.5.1 Differentially expressed gene analysis

**Figure 6:**
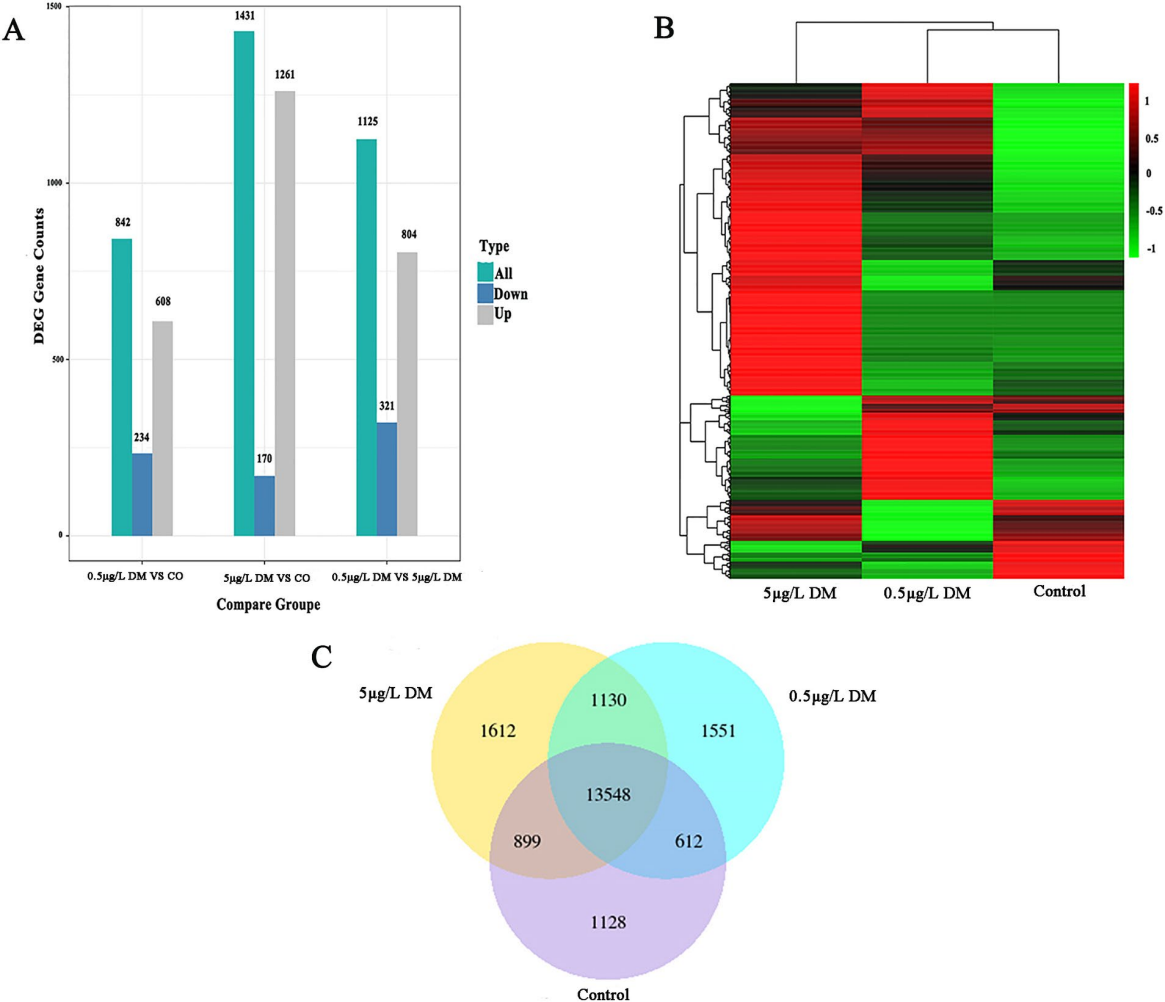
Transcriptome sequencing analysis of the zebrafish deltamethrin (DM) exposure. **(A)** The histogram of the differentially expressed gene (DEG) counts for the control, 0.5 μg/L DM, and 5 μg/L DM groups. The gray bars represent up-regulated genes, the green bars represent all the regulated genes, and the blue dots represent down-regulated genes. **(B)** Heat maps of the control, 0.5 μg/L DM, and 5 μg/L DM groups. The horizontal axis represents the sample group name, and the vertical axis represents the normalized value of the DEG. **(C)** A Venn diagram of the control, 0.5 μg/L DM, and 5 μg/L DM groups.

The differences in the gene expression between the control, 0.5 μg/L DM, and 5 μg/L DM groups are represented by bar plots (Figure 6). When contrasted with the control group, 608 genes were up-regulated and 234 genes were down-regulated in 0.5 μg/L DM group. In comparison, 1,261 genes were up-regulated and 170 genes were down-regulated in 5 μg/L DM group. Through Venn diagram analysis, a total of 13,548 genes were found to be co-expressed in the three groups. These genes may have common functions or participate in common metabolic and signal transduction pathways. In relation to the control group, the gene expression was up-regulated in 5 μg/L DM group. Then, when compared with the genes in 5 μg/L DM group, the expression of most of the genes in 0.5 μg/L DM group was down-regulated.

#### 3.5.2 Gene Ontology functional enrichment analysis of the differentially expressed genes

**Figure 7:**
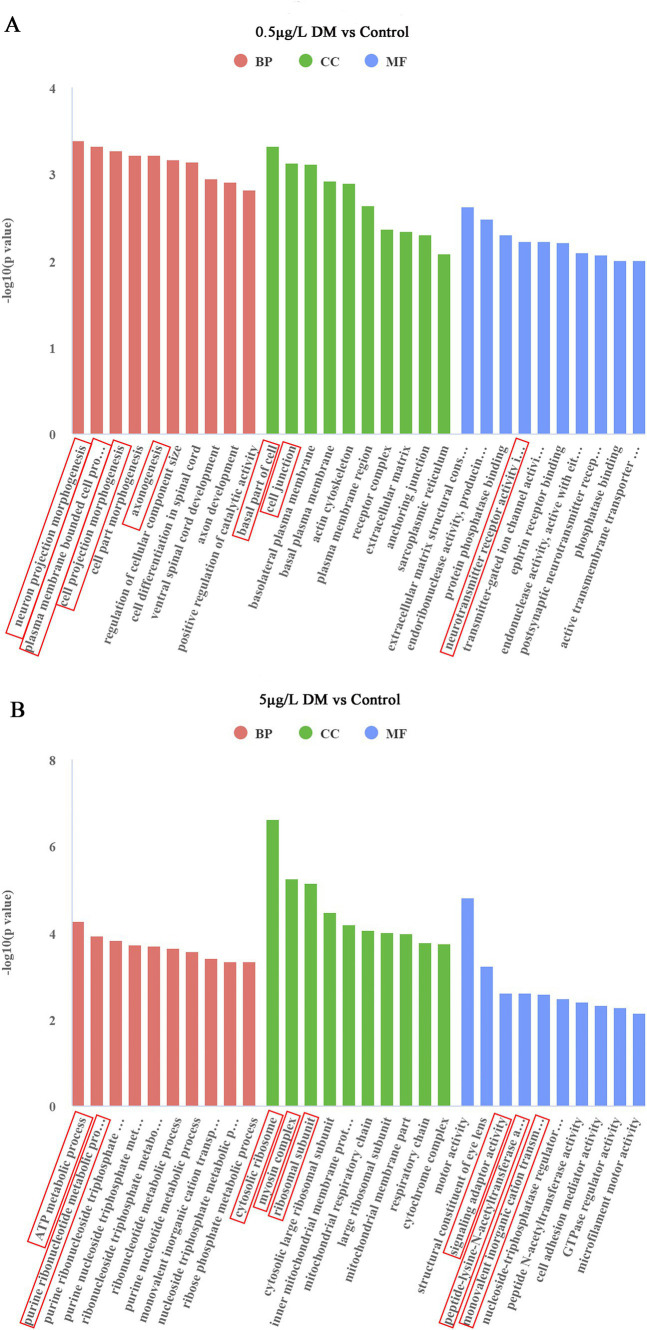
Effect of deltamethrin (DM) exposure on the Gene Ontology (GO) functional enrichment of the differentially expressed genes in zebrafish. **(A)** Comparison between the 0.5 μg/L DM and control groups. **(B)** Comparison between the 5 μg/L DM and control groups. The abscissa indicates the GO terms, the ordinate indicates the significance level of the GO term enrichment, which is expressed as −log 10 (*p*adj), red indicates a biological process (BP), green indicates a cellular component (CC), and blue indicates a molecular function (MF).

The most important 30 pathways among all the pathways that emerged in the GO analysis were selected for the histograms (Figure 7). In comparison with the control group, in 0.5 μg/L DM, the regulation of neuronal projection morphogenesis by the plasma membrane-bounded cells, the generation of cell projection morphogenesis, and axonogenesis were the main biological processes (BPs). The basal part of the cell and cell junctions were enriched in terms of the cellular components (CCs). In addition, the extracellular matrix structural constituent constant endoribonuclease activity products, endoribonuclease activity, production of 5-phosphomonoesterase, protein phosphatase-binding, neurotransmitter receptor activity, adrenoceptor activity, peptidase modulator activity, endonuclease activity, and postsynaptic neurotransmitter reactivity were enriched in terms of molecular function (MF). When compared with the control group, the ATP metabolism process, purine ribonucleotide metabolism, purine nucleoside triphosphate, and ribonucleoside triphosphate metabolism of the BPs were enriched in 5 μg/L DM group; then, the CCs included the cytosolic ribosomes, myosin complexes, and ribosomal subunit; the signal adapter activity, peptide-lysine-N-acetyltransferases, monovalent inorganic cation-responsive receptors, and nucleoside triphosphatase regulatory proteins were enriched in terms of the MF.

#### 3.5.3 Kyoto Encyclopedia of Genes and Genomes analysis of the differentially expressed genes

**Figure 8:**
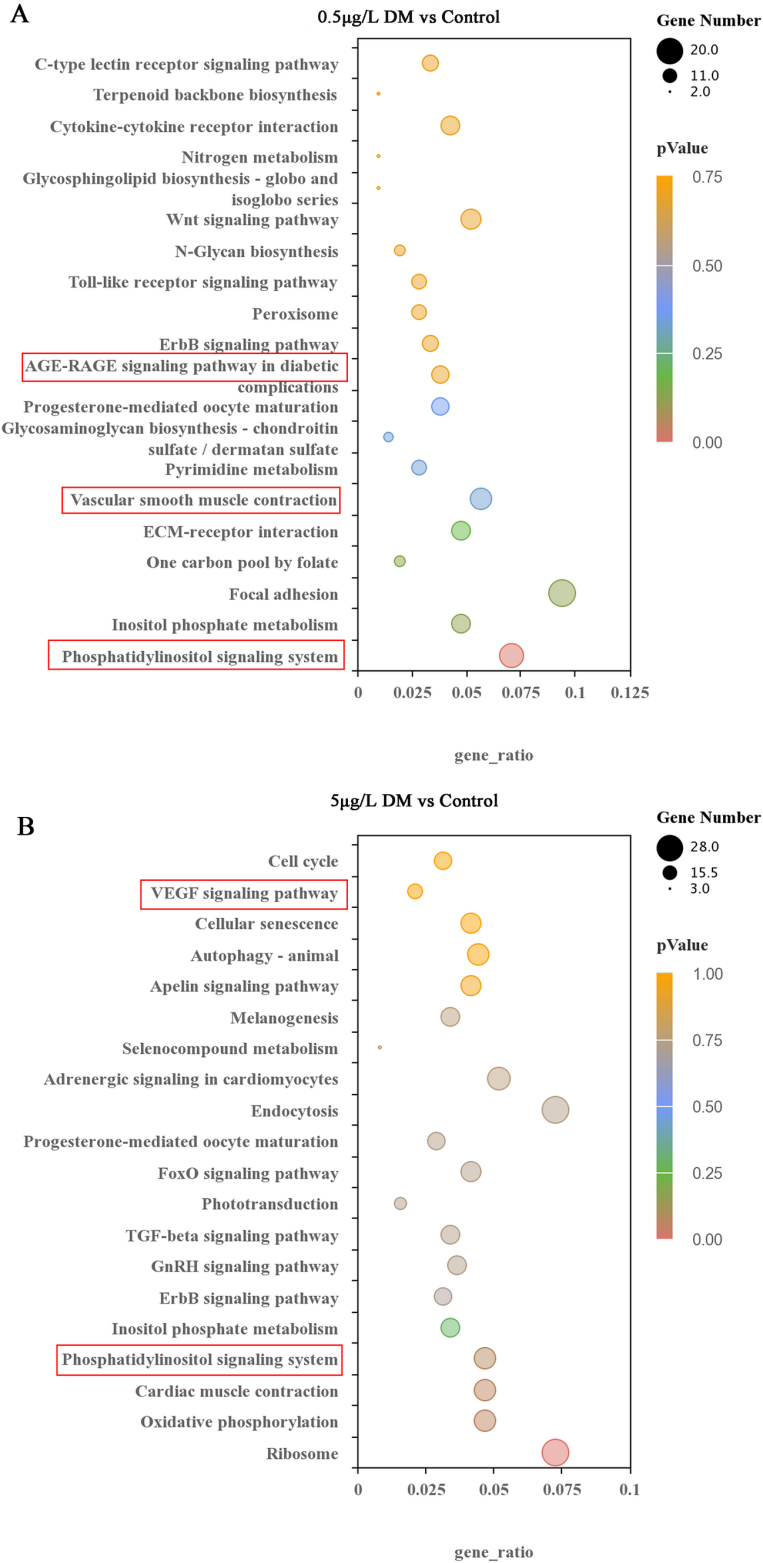
Effects of deltamethrin (DM) exposure on the Kyoto Encyclopedia of Genes and Genomes (KEGG) pathway in zebrafish. The top 20 significantly enriched KEGG pathways are indicated. **(A)** Comparison between the 0.5 μg/L DM and control group. **(B)** Comparison between the 5 μg/L DM and control group. The KEGG nodules containing upregulated genes are marked in blue, and the KEGG nodules containing downregulated genes are marked in green. The circle size indicates the degree of differential gene enrichment. The different colors represent different *p*adj values.

Signaling pathways, such as vascular smooth muscle contraction, the phosphoinositide metabolic pathway, the phosphoinositide signaling system signaling pathway, and the extracellular matrix-receptor interaction signal were enriched in the 0.5 μg/L DM exposure group when contrasted with that of the control group (Figure 8A). In comparison with the control group, the 5 μg/L DM exposure group had an enriched abundant phosphoinositide metabolic pathway and phosphoinositide signaling pathway. However, when compared with the 0.5 μg/L DM exposure group, the 5 μg/L DM exposure group was enriched in the vascular endothelial growth factor signaling pathway and the autophagy and aging cycle pathways (Figure 8B). Moreover, the increase in AGE-RAGE can promote the activation of signaling pathways and cause inflammation and oxidative stress, resulting in cell and tissue damage.

### 3.6 Reverse transcription polymerase chain reaction

**Figure 9:**
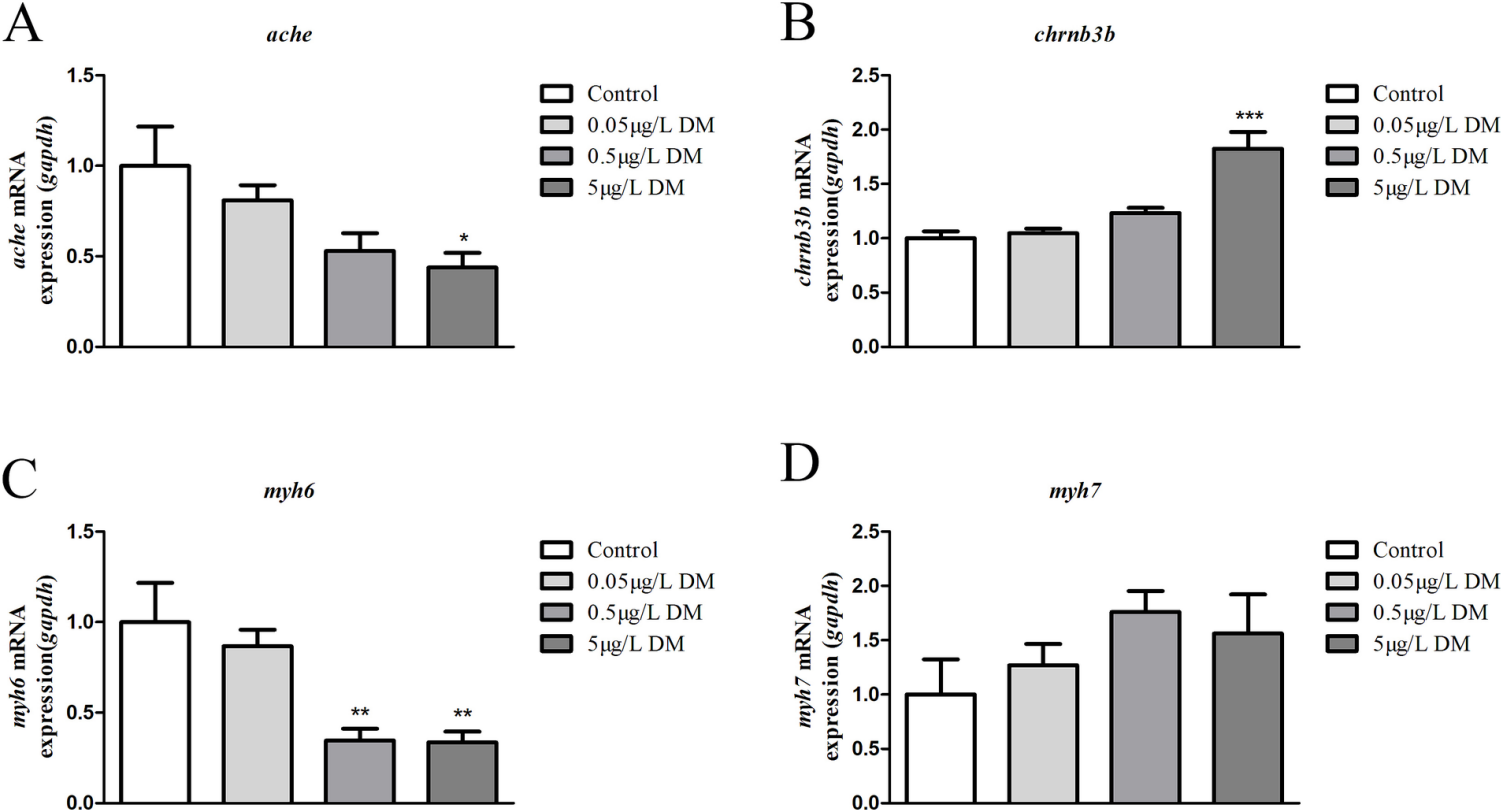
Detection of the gene expression (*ache*, *chrnb3b*, *myh6*, and *myh7* mRNA) in 120 hpf zebrafish larvae that were exposed to deltamethrin (DM). **(A)** *ache* mRNA expression (*: *p* < 0.05). **(B)** *chrnb3b* mRNA expression (***: *p* < 0.05). **(C)** *myh6* mRNA expression (**: *p* < 0.05). **(D)** *myh7* mRNA expression.

The zebrafish embryos were exposed to DM for detection of the AChE gene (*ache*), ACh gene (*chrnb3b*), and vascular smooth muscle associated genes (*myh6* and *myh7*) at 4 hpf to 120 hpf. At 120 hpf, the *ache*, *chrnb3b*, *myh6*, and *myh7* mRNA expression for 0.05 and 0.5 μg/mL DM was compared (Figure 9A). When compared with the control group, 0.05 and 0.5 μg/L DM *ache* mRNA expression decreased by 47.08 and 56.15% (*p* < 0.05; Figure 9A), *chrnb3b* increased by 1.23 times and 1.82 times (*p* < 0.05; Figure 9B), *myh6* mRNA expression decreased by 28.53 and 65.56% (*p* < 0.05), and *myh7* mRNA expression increased by 1.76 times and 1.56 times (*p* > 0.05; Figures 9C,D), respectively.

### 3.7 Acetylcholinesterase activity and acetylcholine content

**Figure 10:**
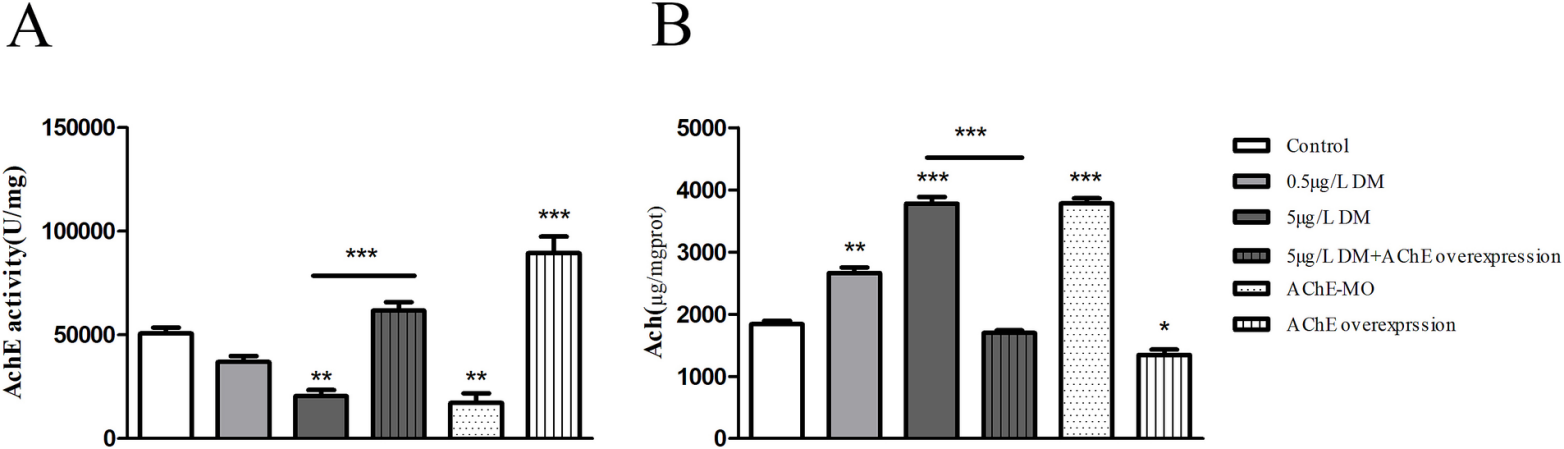
Effects of deltamethrin (DM) exposure on the acetylcholinesterase (AChE) activity, acetylcholine (ACh) content in zebrafish larvae. The zebrafish embryos were exposed to DM from 4 to 120 hpf. **(A)** AChE activity. **(B)** ACh content. (**: *p* < 0.05; ***: *p* < 0.01).

Whether DM exposure affects the changes in AChE activity and the ACh content was determined (Figure 10). When compared with the control group, 0.05 and 0.5 μg/L DM reduced the AChE activity by 27.3 and 59.7% (*p* < 0.05; Figure 10A), respectively. Correspondingly, the ACh content increased significantly in comparison with the control group, increasing by 1.44 and 2.05 times, respectively (*p* < 0.05; Figure 10B).

Zebrafish embryos were injected with AChE mRNA (AChE overexpression) or AChE-MO (AChE knockdown) at the 1–2 cell stage. After DM exposure, the larvae in each group were collected at 120 hpf, and changes in their AChE activities and ACh content were detected. When compared with the control group, AChE-MO reduced the AChE activity by 66.1% (*p* < 0.05), and AChE overexpression increased the AChE activity by 1.76 times (*p* < 0.05). When AChE overexpression was exposed to 0.5 μg/L DM, it reduced the AChE activity by 69.1% (*p* < 0.05). Correspondingly, when AChE overexpression was exposed to 0.5 μg/L DM it was reduced by 20.9% (*p* < 0.05; Figure 10B).

## 4 Discussion

In terms of the acute toxicity of DM, the WHO produced relevant reports in 1990 (25, 26). The LC50 values in fish were confirmed to range from 1.17 μg/L to 33.09 mg/L, but these studies did not include zebrafish. DeMicco et al. (9) reported that the acute toxicity of zebrafish was 40 μg/L and that DM was the most toxic of a variety of pyrethroid pesticides. This was supported by our results. Liu et al. (27) and Chueh et al. (28) also exposed zebrafish to similar concentrations and found that DM caused pericardial edema and functional defects. In this study, similar results were also obtained for pericardial edema. Liu et al. (27) also found that a low concentration of DM (0.025 mg/L) caused changes in the expression levels of sleep/wake genes and hyperactivity in the zebrafish larvae. This was similar to the findings of our study, where 0.05 μg/L DM exposure caused an increase in coiling behavior.

Lei et al. (29) used adult female zebrafish as a model to explore the neurobehavioral changes in the central nervous system that are associated with the environmental levels of DM (30, 100, and 333 ng/L for 21 days). The behavioral measurements showed that DM exposure caused the fish to increase their swimming speed and appear hyperactive. This is similar to our findings, but for mechanistic studies (29), believe that increased exercise is closely related to glutamate and dopamine levels. In this study, the poly-glutamate and dopamine levels were not measured. Both dopamine and ACh are important neurotransmitters in the human body, and they are mutually antagonistic. Under normal circumstances, organisms can function normally by maintaining the balance between dopamine and ACh. When they are out of balance, a series of movement disorders will appear (30).

Acetylcholinesterase is often the object of neurotoxicity studies. Acetylcholinesterase has independently demonstrated its activity in cholinergic neurotransmission during neuronal development (31, 32). However, the role of this factor in axon growth and the maintenance of the neuromuscular system is not fully understood. Some *in vitro* studies have shown that ACh affects neurite elongation by inhibiting neurite growth (33). Thus, one possible role of embryonic AChE would be to neutralize the inhibition of ACh and create a corridor for neurite growth. The cholinergic nervous system is a complex structure with many processes occurring simultaneously and influencing each other (34). In this study, the overexpression and knockout of the AChE gene were investigated in zebrafish embryos using microinjection technology, and they are equivalent to the inhibitors and agonists of AChE. This was conducted to observe the effects of AChE inhibitors and agonists on the behavior of zebrafish and detect the activity of AChE and the ACh content, especially the level of the AChE protein. This can provide a valuable basis for improving the understanding of the role of AChE in behavioral change.

The mechanism of toxicity that is associated with DM is thought to be related to AChE inhibition. Ullah et al. (35) suggested that DM is extremely neurotoxic to fish. Acute exposure to 2 μg/L DM caused a significant decrease in the AChE activity in the brain, liver, and muscle tissue of silver carp (*Hypophthalmichthys molitrix*), resulting in behavioral disorders (35). Although the toxicity of pyrethroid insecticides to birds and mammals is low, DM (20 mg/kg^1^/BW/day) also reduced the activity of cholinesterase after oral administration in laying hens for 14 days (27). This is similar to our results, where 0.15–5 μg/L DM resulted in an enhanced swimming distance and decreased AChE activity in zebrafish. In our study, AChE-MO reduced the AChE activity and increased the swimming speed. Additionally, AChE overexpression increased the AChE activity and reduced the swimming speed. Moreover, when AChE overexpression was exposed to 0.5 μg/L DM, it reduced the AChE activity.

Deltamethrin enters the human body through the food chain and poses a potential threat to the human body’s AChE. Furlong et al. (36) suggested that exposure to pyrethroid insecticides may be associated with the onset of depression in later life. Additionally, von Ehrenstein et al. (37) also reported an increased risk of autism spectrum disorder in offspring after prenatal exposure to environmental pesticides (including polyesters in mothers living within 2,000 m of exposure). Infant exposure further increases the risk of autism spectrum disorder and possible comorbid intellectual disability.

The KEGG enrichment analysis showed that the DEGs were mainly enriched in the Wnt signaling pathway, vascular smooth muscle contraction pathway, phosphoester inositol metabolism, and phosphatidyl inositol pathway. In addition, the combination of the two could promote the activation of the signaling pathway, and eventually cause inflammation and oxidative stress, resulting in cell and tissue damage. Inhibition of AChE due to pathway regulation may be the main factor that is associated with DM-induced behavioral changes in zebrafish. Ahmed et al. (38), in studying the effects on brain function in rats that were exposed to DM, found that DM administration alone resulted in neurobehavioral deficits in rats, manifested as anxiety-like behaviors, and DM significantly inhibited AChE activity. This was similar to the findings of our study. Moreover, we also found that the biological processes that were differentially expressed in the development of DM-exposed zebrafish embryos mainly included synaptic transmission, intercellular signaling, nervous system processes, and other biological processes.

To further explore the molecular mechanism of the toxic effects, RNA-seq analysis was performed to screen for significantly differentially expressed genes and possible pathways. The results showed that DM exposure had significant effects on vascular smooth muscle contraction, the phosphoinositol metabolic pathway, and the apoptosis pathway. Then, Real-Time Quantitative Reverse Transcription PCR was used to verify the validity of the key genes in the pathway. The results showed that DM reduced the gene expressions of AChE and myocardial contraction genes. Thus, the vascular smooth muscle contraction pathway and neuroactive receptor-ligand interaction pathway may lead to neurological dysfunction in zebrafish.

Overall, this study highlights the relationship between behavioral changes and AChE activity in relation to the toxic reactions of zebrafish to DM. We found that DM resulted in increased autonomic activity in early embryos and an increased swimming distance in young zebrafish. We also found that the development of the swim bladder in zebrafish embryos was inhibited with the increase in the DM concentration. Through the mechanism studies, we found that DM inhibited the expression of AChE-related genes and decreased the activity of AChE, which ultimately resulted in behavioral and morphological changes.

## 5 Conclusion

In summary, this study focused on the analysis of zebrafish toxicity responses to DM based on the relationship between behavioral changes and AChE activities. We found that DM resulted in increased coiling movement in the early embryos and increased swimming distance in the zebrafish larvae. Through mechanism research, DM was found to inhibit the expression of AChE-related genes and the activity of the corresponding enzymes. The results of the transcriptome data also showed that low concentration DM induced the ACh-related genes and smooth muscle signaling pathways. Notably, DM induced apoptosis in the zebrafish brain. This indicates that DM exposure may have the potential risk of inducing neurodegeneration, and more attention should be paid to its effects in the future.

## Data Availability

The original contributions presented in the study are included in the article/supplementary material, further inquiries can be directed to the corresponding author/s.
